# Longitudinal Care Cascade Outcomes Among People Eligible for Antiretroviral Therapy Who Are Newly Linking to Care in Zambia: A Multistate Analysis

**DOI:** 10.1093/cid/ciaa268

**Published:** 2020-03-16

**Authors:** Aaloke Mody, David V Glidden, Ingrid Eshun-Wilson, Kombatende Sikombe, Sandra Simbeza, Njekwa Mukamba, Paul Somwe, Laura K Beres, Jake Pry, Carolyn Bolton-Moore, Nancy Padian, Charles B Holmes, Izukanji Sikazwe, Elvin H Geng

**Affiliations:** 1 Division of Infectious Diseases, Washington University School of Medicine, St. Louis, Missouri, USA; 2 Department of Epidemiology and Biostatistics, University of California, San Francisco, San Francisco, California, USA; 3 Centre for Infectious Diseases Research in Zambia, Lusaka, Zambia; 4 Department of International Health, Johns Hopkins University School of Public Health, Baltimore, Maryland, USA; 5 Division of Infectious Diseases, University of Alabama, Birmingham, Alabama, USA; 6 Division of Epidemiology, University of California, Berkeley, Berkeley, California, USA; 7 Department of Medicine, Georgetown University, Washington, D.C., USA

**Keywords:** multistate analysis, HIV care cascade, retention in care, mortality, Zambia

## Abstract

**Background:**

Retention in human immunodeficiency virus (HIV) care is dynamic, with patients frequently transitioning in and out of care. Analytical approaches (eg, survival analyses) commonly used to assess HIV care cascade outcomes fail to capture such transitions and therefore incompletely represent care outcomes over time.

**Methods:**

We analyzed antiretroviral therapy (ART)-eligible adults newly linking to care at 64 clinics in Zambia between 1 April 2014 and 31 July 2015. We used electronic medical record data and supplemented these with updated care outcomes ascertained by tracing a multistage random sample of patients lost to follow-up (LTFU, >90 days late for last appointment). We performed multistate analyses, incorporating weights from sampling, to estimate the prevalence of 9 care states over time since linkage with respect to ART initiation, retention in care, transfers, and mortality.

**Results:**

In sum, 23 227 patients (58% female; median age 34 years [interquartile range 28–41]) were ART-eligible at enrollment. At 1 year, 75.2% had initiated ART and were in care: 61.8% were continuously retained, 6.1% had reengaged after LTFU, and 7.3% had transferred. Also, 10.1% were LTFU within 7 days of enrollment, and 15.2% were LTFU at 1 year (6.7% prior to ART). One year after LTFU, 51.6% of those LTFU prior to ART remained out of care compared to 30.2% of those LTFU after initiating ART. Overall, 6.9% of patients had died by 1 year with 3.0% dying prior to ART.

**Conclusion:**

Multistate analyses provide more complete assessments of longitudinal HIV cascade outcomes and reveal treatment gaps at distinct timepoints in care that will still need to be addressed even with universal treatment.

## Teaser

Multistate analyses that account for patient transitions across human immunodeficiency virus (HIV) care cascade steps over time can provide more complete depictions of longitudinal treatment outcomes and highlight care gaps that will still need to be addressed even with universal HIV treatment.

Despite rapid expansion of antiretroviral therapy (ART) coverage in sub-Saharan Africa over the past decade, human immunodeficiency virus (HIV) treatment outcomes such as viral suppression and mortality still remain suboptimal [1]. Successful treatment requires a series of steps—which include linkage to HIV care, ART initiation, and retention—but significant care gaps exist at each step of this HIV care cascade. Although previous studies have highlighted that patients frequently transition back and forth between care cascade steps over time [2–8], current approaches to understanding these treatment gaps still frequently approach the cascade as a linear sequence of events [2–10]. These approaches either represent the care cascade as a series of cross-sectional estimates or use longitudinal methods to examine only 1 cascade step at a time. By overlooking patient transitions into and out of different care states over time (ie, retained vs not retained, on ART vs not on ART), these assessments may provide incomplete representations of the actual longitudinal experience of patients receiving HIV care [2–10].

Multistate analytic approaches can account for patient transitions in between different care states over time and may better characterize patients’ care and treatment experience [11–13]. By highlighting the combined dynamics of multiple cascade steps, these approaches provide more complete and nuanced representations of longitudinal patient outcomes and can more precisely characterize the remaining care gaps. For example, in the current era of universal treatment, the question remains as to whether rapidly initiating ART actually improves downstream outcomes. Traditional approaches to the care continuum typically will examine effects on either ART initiation or retention in isolation, but multistate approaches can extend these insights by also identifying whether early loss to follow-up (LTFU) simply shifts from pre- to post-ART or whether failures to initiate ART are driven by patients becoming LTFU or provider-level delays. This type of comprehensive assessment of patient cares states over time can thus better characterize the persistent treatment gaps and, ultimately, inform how to best target and implement interventions to improve the HIV care cascade [5–10].

In this analysis, we use multistate analytic techniques to examine longitudinal care experience among patients eligible for ART newly linking to HIV care in Zambia in a way that accounts for several key transitions along the HIV care cascade, including ART initiation, transitions into and out of retention in care, transfers to new clinics, and death.

## METHODS

### Patient Population and Setting

We analyzed a cohort of ART-naiive adults (>18 years old) with HIV who newly enrolled in care between 1 April 2014 and 31 July 2015 and who were eligible for ART according to Zambian national treatment guidelines at the time of enrollment (ie, a CD4 count <500 cells/μL, active tuberculosis, World Health Organization [WHO] clinical stage 3 or 4 disease, or pregnancy or breastfeeding [14]). Patients were from 64 Zambian Ministry of Health clinics that received technical support from the Centre for Infectious Disease Research in Zambia (CIDRZ), a nongovernmental organization operating across 4 of the 10 provinces in Zambia.

### Measurements

Sociodemographic, clinical, facility-level, and visit history measurements were obtained from the national electronic medical record (EMR) system used in routine HIV care in Zambia. To populate the EMR, providers first complete standardized paper clinical forms during routine patient encounters, and then data clerks enter this information into the electronic database. Additionally, we undertook a multistage sampling-based approach in order to obtain accurate population-representative estimates of retention and mortality (S1 appendix) [15–17]. We first took a stratified random sample of 32 of the total 64 facilities, and then, at selected facilities, we enumerated a random sample of patients who were LTFU as of 31 July 2015 (defined as being at least 90 days late for the last scheduled visit or more than 180 days without any visit based on guidelines at the time [18]). Selected patients were then traced using a combination of chart review, phone calls, and in-person visits within the community, and we used structured questionnaires to record their current vital and care status and associated dates.

### Analyses

**Figure 1.**
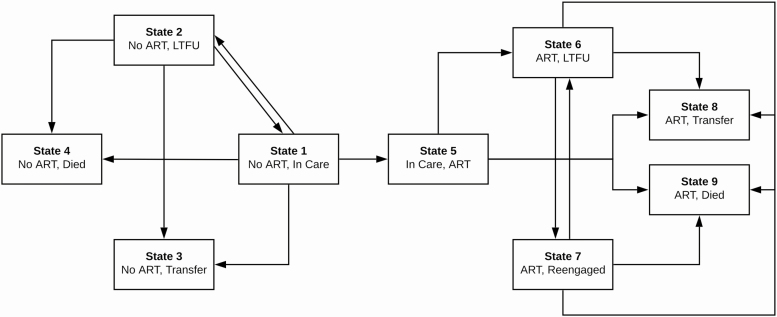
State transitions framework for multistate analysis. At each time point, patients were categorized into 1 of 9 mutually exclusive and exhaustive states: (1) not initiated on ART and in care, (2) out of care prior to initiating on ART, (3) transfer to another clinic prior to initiating ART, (4) death prior to initiating ART, (5) initiated on ART and consistently in care (ie, no LTFU after initiating ART), (6) out of care after initiating ART, (7) reengaged in care at the original clinic after LTFU on ART, (8) transfer to another clinic after initiating ART, and (9) death after initiating ART. This figure depicts all the possible transitions patients could make from each state. Abbreviations: ART, antiretroviral therapy; LTFU, lost to follow-up.

We sought to describe the longitudinal experience of patients newly enrolling in HIV care in a manner that accounted for transitions between ART states (eg, initiated on ART or not) and retention states (eg, in care, LTFU, reengaged after LTFU, transferred) over time [11–13]. To do so, we used multistate analytic techniques to estimate the probability of a patient being in a particular care state at any given time point since enrollment. We first categorized patients at each time point into 1 of 9 mutually exclusive and exhaustive states: (1) not initiated on ART and in care, (2) out of care prior to initiating ART, (3) transfer to another clinic prior to initiating ART, (4) death prior to initiating ART, (5) initiated on ART and consistently in care (ie, no LTFU after initiating ART), (6) out of care after initiating ART, (7) reengaged in care at the original clinic after LTFU on ART, (8) transfer to another clinic after initiating ART, and (9) death after initiating ART (Figure 1). We then applied nonparametric multistate analytic techniques based on the Aalen-Johansen method that are designed to account for patient transitions into and out of multiple nonabsorbable states over time [11–13, 19]. Time zero was the enrollment date, and patients were censored at the time of transfer, death, or the end of the observation period (ie, 31 July 2015). In addition, we performed separate analyses to assess outcomes specifically among patients after ART initiation or LTFU from care; time zero for these analyses was the entry date into that particular state. Finally, we performed stratified analyses among patient subgroups, reporting the proportion of patients in each of 4 composite states at 1 year since enrollment: (1) in care and on ART (a composite of ART in care [state 5], reengaged on ART [state 7], and transferred on ART [state 8]), (2) LTFU, (3) transfer to a new clinic, and (4) died. All analyses incorporated sampling weights based on the inverse of the probability of having outcomes successfully ascertained from the multistage sampling; weights were recalibrated for subgroups in stratified analyses (S1 appendix) [20]. We used bootstrapping (n = 1000 iterations) to obtain 95% confidence intervals. Analyses were conducted using the *mstate* package in R [12, 13].

Finally, we also used Cox proportional-hazards models to identify baseline characteristics independently associated with LTFU, transfer, and death both prior to and after ART initiation. We used multiple imputation (n = 20) to address missingness in predictor variables [15, 16].

## RESULTS

### Patient Characteristics

**Figure 2.**
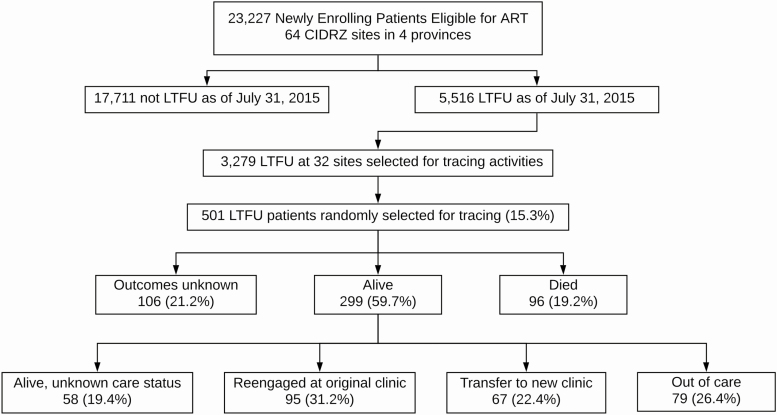
Patient flowchart. In sum, 23 227 patients eligible for ART at the time of enrollment at 64 sites were included in this analysis. As of 1 July 2015, 5516 were considered LTFU, and we randomly selected 501 patients from 32 sites for active tracing. We ascertained updated vital status in 395 (78.8%) of the patients and updated care status in 241 of the 299 patients known to be alive (80.6%). Abbreviations: ART, antiretroviral therapy; LTFU, lost to follow-up.

During the study period, 23 227 people eligible for ART newly enrolled in HIV care at one of 64 clinics across 4 provinces in Zambia (Figure 2). Most were female (58.0%), with a median age of 34 years (interquartile range [IQR] 28–41) and median enrollment CD4 count of 268 cells/µL (IQR 134–430). We attempted to trace 501 LTFU patients between September 2015 and July 2016 and successfully ascertained updated vital and care status in 337 (67.3%). Sampled patients and those successfully traced were mostly similar in baseline characteristics, although we were less successful in tracing younger patients and those from Lusaka province (Table 1).

**Table 1.** Baseline Patient Characteristics, N = 23 227

	All Patients (N = 23 227)	Lost (n = 5516)	Sampled (n = 501)	Successfully Traced (n = 337)
Sex, n (%)				
Male	9764 (42.0%)	2418 (43.8%)	225 (44.9%)	158 (46.9%)
Female	13 463 (58.0%)	3098 (56.2%) |	276 (55.1%)	179 (53.1%)
Enrollment age, n (%)				
<25 y	2885 (12.4%)	840 (15.2%)	66 (13.2%)	36 (10.7%)
25–35 y	9281 (40.0%)	2351 (42.6%)	198 (39.5%)	128 (38.0%)
35–50 y	9006 (38.8%)	1907 (34.6%)	201 (40.1%)	144 (42.7%)
>50 y	2055 (8.8%)	418 (7.6%)	36 (7.2%)	29 (8.6%)
Enrollment CD4 count (cells/μL), n (%)				
<200	9234 (39.8%)	2248 (40.8%)	207 (41.3%)	146 (43.3%)
200–350	6321 (27.2%)	1284 (23.3%)	100 (20.0%)	61 (18.1%)
350–500	4557 (19.6%)	979 (17.7%)	93 (18.6%)	65 (19.3%)
>500	1371 (5.9%)	468 (8.5%)	39 (7.8%)	24 (7.1%)
Unknown	1744 (7.5%)	537 (9.7%)	62 (12.4%)	41 (12.2%)
Enrollment WHO stage, n (%)				
I	9941 (42.8%)	2113 (38.3%)	189 (37.7%)	123 (36.5%)
II	3977 (17.1%)	784 (14.2%)	78 (15.6%)	50 (14.8%)
III	6107 (26.3%)	1784 (32.3%)	139 (27.7%)	95 (28.2%)
IV	505 (2.2%)	172 (3.1%)	26 (5.2%)	16 (4.7%)
Unknown	2697 (11.6%)	663 (12.0%)	69 (13.8%)	53 (15.7%)
Marital status, n (%)				
Single	2527 (10.9%)	694 (12.6%)	70 (14.0%)	53 (15.7%)
Married	11 965 (51.5%)	2798 (50.7%)	272 (54.3%)	190 (56.4%)
Divorced	2901 (12.5%)	751 (13.6%)	54 (10.8%)	36 (10.7%)
Widowed	1621 (7.0%)	331 (6.0%)	36 (7.2%)	24 (7.1%)
Unknown	4213 (18.1%)	942 (17.1%)	69 (13.8%)	34 (10.1%)
Education, n (%)				
None	1494 (6.4%)	345 (6.3%)	39 (7.8%)	23 (6.8%)
Lower-mid basic	6926 (29.8%)	1579 (28.6%)	181 (36.1%)	132 (39.2%)
Upper basic/secondary	9872 (42.5%)	2521 (45.7%)	202 (40.3%)	129 (38.3%)
College/university	897 (3.9%)	183 (3.3%)	26 (5.2%)	22 (6.5%)
Unknown	4038 (17.4%)	888 (16.1%)	53 (10.6%)	31 (9.2%)
Facility type, n (%)				
Urban	14 259 (61.4%)	3927 (71.2%)	255 (50.9%)	160 (47.5%)
Rural	1610 (6.9%)	420 (7.6%)	122 (24.4%)	100 (29.7%)
Hospital	7358 (31.7%)	1169 (21.2%)	124 (24.8%)	77 (22.8%)
Province, n (%)				
Lusaka	13 177 (56.7%)	3901 (70.7%)	265 (52.9%)	154 (45.7%)
Eastern	4446 (19.1%)	586 (10.6%)	75 (15.0%)	55 (16.3%)
Southern	2573 (11.1%)	448 (8.1%)	102 (20.4%)	77 (22.8%)
Western	3031 (13.0%)	581 (10.5%)	59 (11.8%)	51 (15.1%)

Abbreviation: WHO, World Health Organization.

### Longitudinal Care Cascade Outcomes From Multistate Analyses

**Figure 3.**
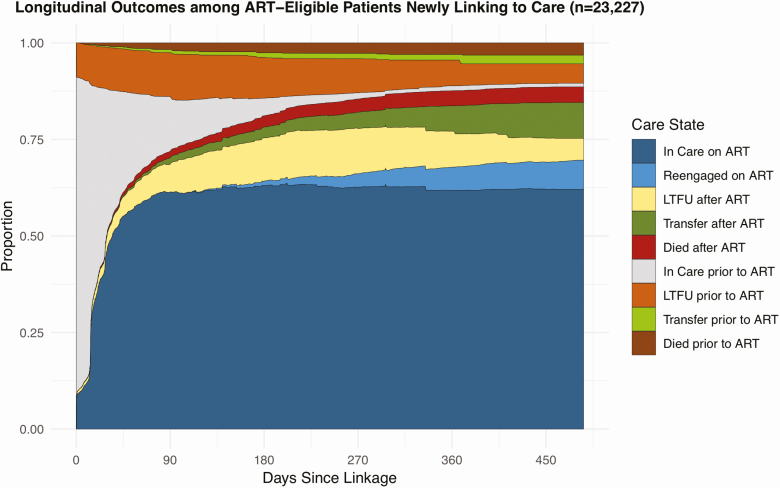
Longitudinal outcomes among people eligible for ART newly linking to care.This figure represents longitudinal outcomes among 23 227 people eligible for ART newly linking to care in Zambia based on results from the overall multistate analysis. The figure depicts the proportion of patients estimated to be in each care state at any given time point accounting for the transitions patients made between different care states over time. Abbreviations: ART, antiretroviral therapy; LTFU, lost to follow-up.

Among people eligible for ART at the time of enrollment, 71.9% had initiated ART by 3 months, and 87.6% had initiated ART by 12 months. At 1 year, 75.2% of patients had initiated ART and were still in care: 61.8% had remained consistently retained at the original facility; 6.1% were reengaged after previously experiencing LTFU; and 7.3% had transferred to another clinic. Only 1.3% of patients were in care but had not yet initiated ART. Also, 10.1% of patients dropped out of care within 7 days of enrollment, and 15.2% were out of care at 1 year, with 6.7% being out of care without ever initiating ART and the remaining 8.5% out of care after initiating ART. Overall, 6.9% of patients had died by 1 year with 3.0% dying prior to initiating ART and 3.9% dying after initiating ART (Figure 3, Table S1).

**Figure 4.**
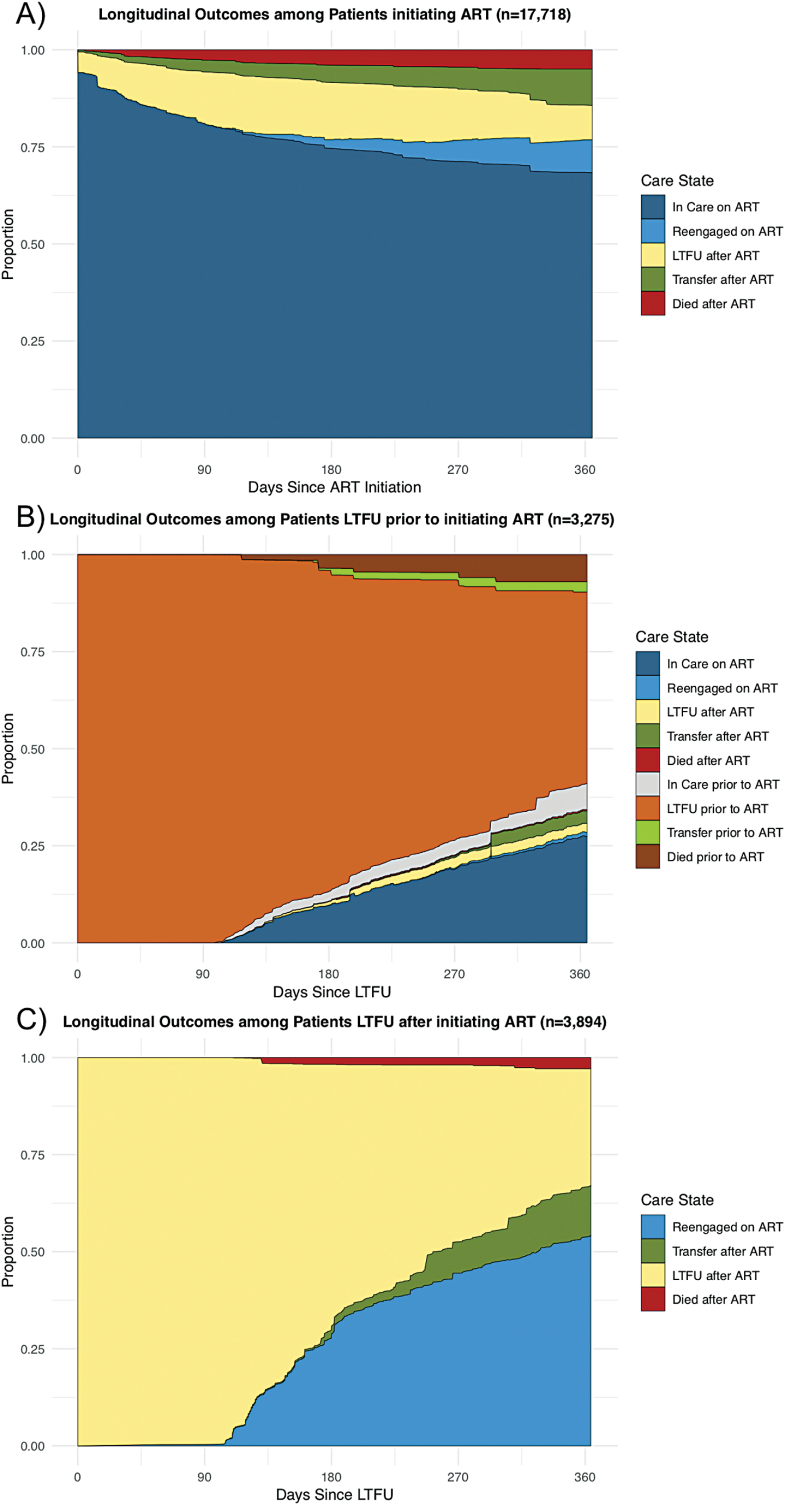
Longitudinal outcomes among patients newly entering a specific care state. These figures represent longitudinal outcomes among the patients after newly entering 3 specific care states. *A,* Outcomes after patients initiate ART. *B*, Outcomes after patients become LTFU prior to initiating ART. *C*, Outcomes after patients become LTFU after previously initiating ART. Abbreviations: ART, antiretroviral therapy; LTFU, lost to follow-up.

Among people who initiated ART, 86.2% overall were in care and on ART 1 year after initiating ART (68.3% continuously in care, 8.5% reengaged after LTFU, and 9.4% transferred), 8.8% of patients were LTFU, and 5.0% had died. Among the LTFU, reengagement substantially differed depending on whether LTFU occurred prior to or after initiating ART. At 1 year after becoming LTFU prior to ART, only 31.7% of patients were in care and initiated on ART, whereas 51.6% remained LTFU, 5.8% transferred to another clinic, and 7.4% died. In contrast, among those who were LTFU after initiating ART, 66.9% were in care and on ART, 30.2% were still LTFU, 12.9% had transferred, and 2.8% had died (Figure 4, Table S2).

**Figure 5.**
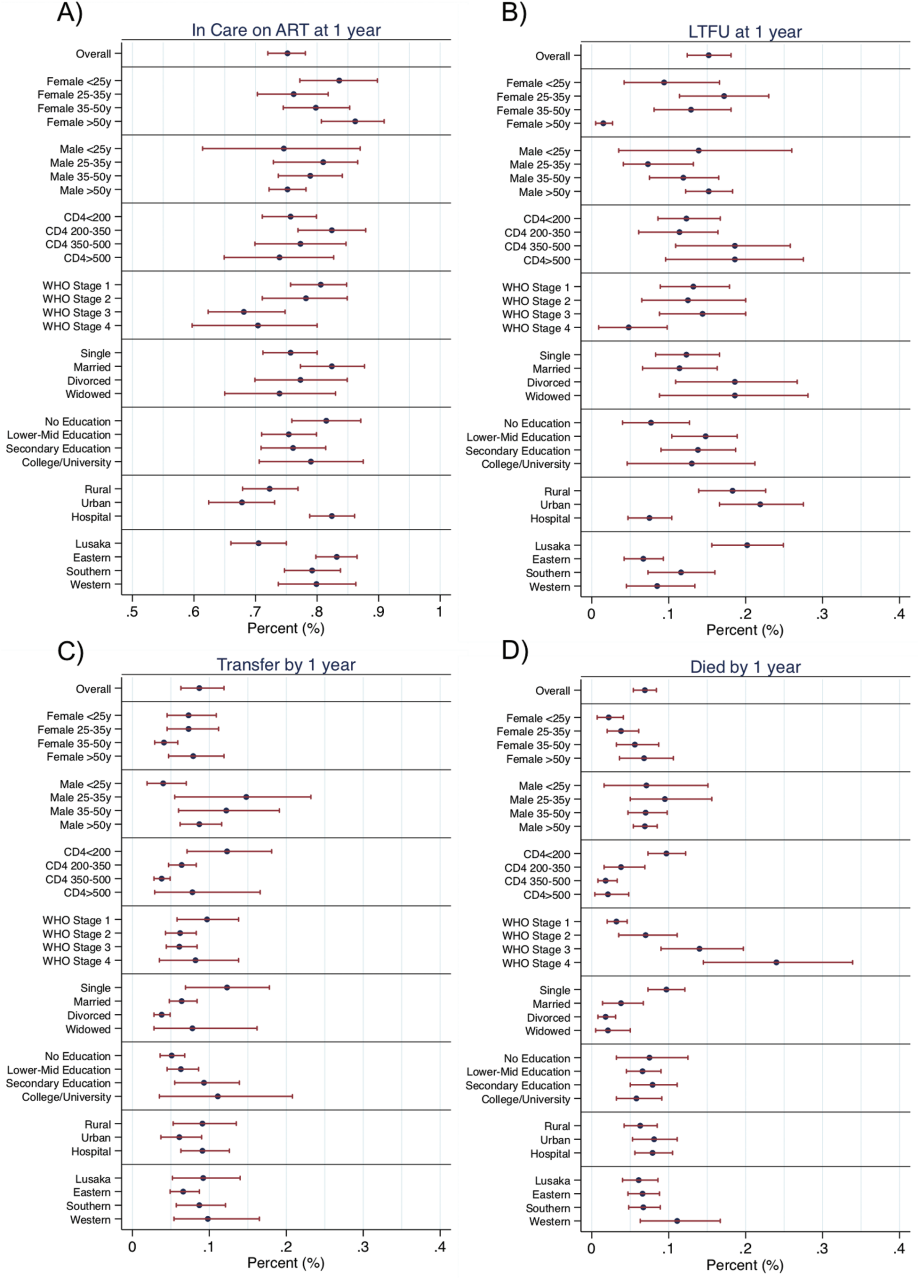
Forest plot of results from multistate analyses stratified by patient subgroups. This figure depicts the proportion of patients in 1 of 4 composite care states at 1 year after performing stratified multistate analysis. *A*, Proportion in each subgroup who are in care and on ART (a composite of ART in care [state 5], reengaged on ART [state 7], and transferred on ART [state 8]). *B*, Proportion of each subgroup who are LTFU (a composite of LTFU prior to ART [state 2] and LTFU after ART [state 6]). *C*, Proportion in each subgroup who transferred to a new clinic (a composite of transfer prior to ART [state 3] and transfer after ART [state 8]). *D*, Proportion who have died (a composite of died prior to ART [state 4] and died after ART [state 9]). Abbreviations: ART, antiretroviral therapy; LTFU, lost to follow-up.

In subgroup analyses, women >50 years old, patients attending a hospital-based clinic, and patients not from Lusaka province were most likely to be in care and on ART at 1 year. In contrast, patients with higher enrollment CD4s, divorced or widowed patients, patients not attending a hospital-based clinic, and those from Lusaka province had a higher proportion of out of care at 1 year. The patients most likely to transfer to a new facility were men between the age of 25 and 50, women over 50, single patients, and those with more education. Finally, those most likely to die included older women, men irrespective of age, those with lower CD4 and higher WHO stage, single patients, and patients from Western province (Figure 5, Table S3).

### Factors Associated With LTFU, Transfer, and Death Prior to and After ART Initiation

In multivariable regression, LTFU prior to ART was associated with a higher CD4 and being from Lusaka, whereas LTFU after ART was associated with younger age, being married, and not attending a hospital-based facility. Patients who were men, not married, and less sick (ie, higher CD4 count and lower WHO stage) were more likely to transfer prior to ART, whereas those who were men, older, single, and sicker (ie, lower CD4 count and higher WHO stage) were more likely to transfer after ART. Finally, lower CD4 count (but not higher WHO stage) was associated with dying prior to ART, whereas older age, higher WHO stage (but not lower CD4 count), and being from a rural area (ie, not Lusaka) were more likely to die after initiating ART (Table 2).

**Table 2.** Cox Proportional Hazards Regression of Factors Associated with LTFU, Transfer, and Death Prior to and After ART Initiation, N = 23 227

	LTFU				Transfer				Died			
	Prior to ART		After ART		Prior to ART		After ART		Prior to ART		After ART	
	aHR (95% CI)	*P* Value	aHR (95% CI)	*P* Value	aHR (95% CI)	*P* Value	aHR (95% CI)	*P* Value	aHR (95% CI)	*P* Value	aHR (95% CI)	*P* Value
Male sex	1.02 (.72–1.44)	.91	0.94 (.68–1.29)	.68	1.49 (.54–4.14)	.44	1.58 (1.08–2.31)	.019	1.11 (.53–2.32)	.79	0.89 (.56–1.41)	.63
Age, per 10 y increase	0.95 (.83–1.08)	.41	0.78 (.68–.90)	.001	1.27 (.84–1.91)	.25	1.17 (.99–1.38)	.071	0.97 (.72–1.30)	.84	1.62 (1.44–1.82)	<.001
Enrollment CD4 count, per 100 cells/μL decrease	0.86 (.83–.90)	<.001	1.02 (.94–1.11)	.66	0.78 (.66–.91)	.002	1.20 (1.01–1.43)	.044	2.41 (1.56–3.74)	<.001	1.24 (1.04–1.48)	.019
WHO stage												
I	Ref	.74	Ref	.48	Ref	.040	Ref	.0035	Ref	.72	Ref	<.001
II	1.02 (.63–1.65)		1.10 (.69–1.76)		1.29 (.35–4.71)		0.60 (.34–1.04)		1.06 (.32–3.51)		2.06 (1.01–4.20)	
III	1.21 (.81–1.81)		0.82 (.57–1.19)		0.32 (.13–.81)		0.68 (.34–1.34)		1.46 (.62–3.46)		3.06 (1.61–5.83)	
IV	0.73 (.18–2.99)		1.46 (.57–3.76)		0.31 (.06–1.77)		2.95 (1.30–6.68)		1.91 (.51–7.25)		10.95 (4.79–25.01)	
Marital status												
Single	0.92 (.59–1.43)	.96	0.72 (.42–1.24)	.019	4.05 (1.07–15.33)	.004	2.97 (1.51–5.84)	<.001	1.26 (.50–3.21)	.43	2.44 (1.19–5.03)	.12
Married	Ref		Ref		Ref		Ref		Ref		Ref	
Divorced	0.92 (.56–1.51)		0.50 (.31–.79)		5.50 (2.08–14.55)		0.47 (.31–.72)		0.58 (.21–1.56)		0.98 (.38–2.54)	
Widowed	0.90 (.47–1.73)		0.69 (.38–1.23)		1.39 (.49–3.92)		0.86 (.56–1.33)		1.46 (.59–3.62)		1.24 (.60–2.55)	
Education												
None	Ref	.009	Ref	.34	Ref	.22	Ref	.103	Ref	.86	Ref	.074
Lower-mid basic	1.39 (.79–2.45)		0.85 (.55–1.31)		1.59 (.62–4.04)		1.19 (.72–1.99)		1.46 (.47–4.48)		1.78 (1.04–3.03)	
Upper basic/secondary	0.76 (.41–1.38)		0.92 (.61–1.39)		0.66 (.15–2.82)		1.56 (1.01–2.41)		1.18 (.32–4.31)		2.08 (1.10–3.93)	
College/university	1.23 (.53–2.83)		1.53 (.78–3.03)		2.84 (.65–12.45)		2.08 (.70–6.16)		0.84 (.12–5.73)		3.23 (.90–11.62)	
Facility type												
Urban	Ref	.12	Ref	<.001	Ref	.40	Ref	.11	Ref	.37	Ref	.023
Rural	1.28 (.90–1.83)		0.95 (.68–1.34)		0.62 (.26–1.51)		0.87 (.49–1.57)		0.66 (.33–1.30)		1.58 (.89–2.79)	
Hospital	0.85 (.56–1.29)		0.44 (.30–.63)		0.62 (.23–1.65)		1.43 (.97–2.13)		0.70 (.36–1.37)		0.82 (.46–1.44)	
Province												
Lusaka	Ref	.03	Ref	.086	Ref	.014	Ref	.37	Ref	.58	Ref	.004
Eastern	0.56 (.37–.85)		1.11 (.80–1.53)		0.95 (.38–2.36)		0.75 (.50–1.12)		0.61 (.27–1.38)		2.95 (1.50–5.82)	
Southern	0.99 (.69–1.42)		0.81 (.55–1.19)		0.87 (.32–2.32)		0.83 (.47–1.45)		0.74 (.37–1.46)		2.24 (1.12–4.48)	
Western	0.60 (.32–1.11)		1.53 (.98–2.39)		0.20 (.06–.66)		1.11 (.65–1.91)		1.19 (.50–2.83)		3.25 (1.63–6.49)	

All estimates are from multistate models.

Abbreviations: aHR, adjusted hazard ratio; ART, antiretroviral therapy; CI, confidence interval; LTFU = lost to follow-up; Ref, reference value; WHO, World Health Organization.

## DISCUSSION

Using multistate analytic methods, we characterized the longitudinal experience of ART-eligible patients newly enrolling in HIV care accounting for the transitions between different care states that a patient may make over time. After 1 year, 75.2% of patients had initiated ART and were in care overall; 61.8% had remained consistently retained, 6.1% were reengaged after LTFU, and 7.3% had transferred to another clinic. In addition, 10.1% of patients became LTFU within 7 days of enrollment, and 15.2% of patients were out of care at 1 year with 6.7% out of care prior to even initiating ART. Overall, 6.9% of patients had died by 1 year with 3.0% dying prior to initiating ART. Among the LTFU, reengagement by 1 year substantially differed depending on whether LTFU occurred prior to (51.6% remained LTFU) or after initiating ART (30.2% remained LTFU). Overall, these findings provide a comprehensive depiction of the HIV care experience over time among patients eligible for ART at the time of enrollment and highlights the importance accounting for care transitions when examining longitudinal outcomes along the HIV care cascade.

Our study demonstrates that early disengagement from care has potential to limit the impact of current policies for universal treatment if not adequately addressed. Among people who were eligible for ART, failure to start ART was primarily concentrated in patients who became LTFU early on—a majority of whom did so after only 1 visit to the clinic—with very few patients who remained in care not being initiated on ART. It is unclear whether rapid ART initiation efforts will simply shift early LTFU from prior to ART initiation to post-ART initiation in real-world settings, potentially limiting their impact on longer-term outcomes [21–29]. In our study, 43.5% of all mortality occurred in patients prior to ART initiation, and 48.2% of these patients had at least a 30-day lapse in care prior to their death. Although it is unclear in whom ART would have altered these outcomes, the effects of rapid and universal ART initiation may be blunted if they do not occur in tandem with sustained retention after linkage. Thus, effective strategies for rapidly cultivating meaningful engagement beyond the initial visit are needed, and metrics for early treatment success should integrate both ART initiation and early retention to be most relevant for the contemporary era [30, 31].

Current retention strategies generally treat LTFU as a uniform entity [32], but our results indicate that understanding the mechanisms of LTFU is essential for improving their effectiveness. We found that patients who become LTFU prior to ART initiation are much less likely to reengage in care or transfer compared to those who become LTFU after initiating ART. We posit that these differences are likely driven by the underlying mechanisms associated with becoming LTFU at different time points (ie, early in care prior to starting ART vs after engaging in care long enough to be initiated on ART), rather than simply being an effect of initiating ART. Previous work on patient-reported reasons for LTFU—including latent class analyses that identified unique LTFU phenotypes—support this hypothesis that barriers to care and care seeking behaviors vary across different stages of treatment [33–37]. Thus, future retention interventions should attempt to leverage patients’ treatment histories in order to more effectively target different mechanisms of LTFU.

Beyond the initial treatment period, our study also identified important downstream treatment gaps in the HIV care cascade. We found that only 61.8% of patients remained continuously retained after ART initiation over the first year, whereas an additional 13.4% were in care only after reengaging after LTFU or transferring to a new clinic. Patients frequently churn in and out of care over time [2–8], but these transitions are often also associated with prolonged lapses in treatment [4]. Thus, they represent unique opportunities to intervene in patients who have already demonstrated they are at risk for poor engagement. For example, Medecins Sans Frontieres’ “Welcome Back” program proactively facilitates care for returning patients in order to strengthen engagement and mitigate patient fears of poor treatment after their care lapse [36–38]. Streamlining the process of transferring between facilities could also help to minimize associated treatment lapses [32]. Finally, differentiated service delivery can reduce the burden of accessing care for the majority of patients who remain stable while also reducing patient volumes and allowing resources to be more focused on those who need it most [32]. Thus, understanding the heterogeneity in patients’ treatment histories over time can help treatment programs develop more comprehensive care delivery packages and move beyond 1-size-fits-all approaches.

Novel longitudinal methods such as multistate analyses represent a promising but underutilized tool for monitoring and evaluation as they can capture the dynamic nature of outcomes along HIV care cascade over time [5–8]. Patients’ treatment journeys do not linearly progress through the care cascade 1 step at time. Rather, patients commonly transition back and forth between multiple care states over time, and these methods allow us to better quantify outcomes in relation to multiple interlinked cares states such as ART initiation, retention in care, LTFU, and reengagement over time. These methods are gaining traction [5–8, 11–13], but their use should be extended to other outcomes that commonly fluctuate over time, both in HIV (eg, viral load monitoring [suppressed vs unsuppressed or up to date vs not up to date], or pre-exposure prophylaxis persistence) as well as noncommunicable diseases (eg, medication and control status for hypertension or diabetes). Compared to more typical approaches (eg, cross-sectional analyses or survival analyses focused on single outcomes), these methods provide a better and more comprehensive approach to measuring patient outcomes along care cascades over time.

There are several limitations to this study. First, we were less successful in tracing younger patients and patients from Lusaka, although we attempted to minimize any bias from this by accounting for differences in tracing success in our sampling weights. Second, we were unable to follow patients after they had transferred to a different facility. Thus, we could not capture care transitions that occurred after transfer, although previous work has documented delays in ART reinitiation among those transferring without official documentation [4]. Third, treatment standards have changed (ie, implementation of universal treatment) since our data were collected in 2015 [14]. Nevertheless, our cohort represents patients who were eligible for ART at the time of linkage, and it is likely that the general patterns we observed (except for the rapidity of ART initiation) will still be relevant in the current era. Fourth, we had a limited duration of follow-up, precluding assessments of longer-term outcomes such as what happens after patients reengage in care after LTFU. Finally, we were unable to assess virologic outcomes as viral loads were not routinely collected in Zambia during our study period, although results from the ZAMPHIA survey suggest that just under 90% of patients in care and on ART will be virally suppressed [39].

In conclusion, we used multistate analytic methods to fully characterize longitudinal outcomes along the HIV care cascade among people eligible for ART newly linking to care in Zambia. We found that 75.2% of patients overall were in care and on ART at 1 year, but only 61.8% had remained consistently retained. Despite being ART-eligible, 43.5% of all mortality occurred in patients prior to even initiating ART with failure to initiate ART primarily being driven by early lapses in care. Finally, we found that the likelihood of returning to care after LTFU differed depending on whether LTFU occurred prior to versus after ART initiation. Multistate approaches help to reveal the distinct care gaps at distinct time points that ultimately limit sustained treatment success, highlighting the importance of examining HIV care cascade outcomes in a manner that accounts for care transitions that occur over the course of a patient’s treatment history. Future efforts must now focus on learning how to best implement comprehensive packages of care that effectively target the varied care challenges patients may face over the course of lifelong treatment.

## Supplementary Data

Supplementary materials are available at *Clinical Infectious Diseases* online. Consisting of data provided by the authors to benefit the reader, the posted materials are not copyedited and are the sole responsibility of the authors, so questions or comments should be addressed to the corresponding author.

ciaa268_suppl_Supplementary_Appendix

## Notes

***Acknowledgments.*** The authors thank all staff at the Centre for Infectious Diseases Research in Zambia (CIDRZ). They also thank the Zambian Ministry of Health, Center for Disease Control and Prevention, and President’s Emergency Plan For AIDS Relief (PEPFAR) for developing the health systems and electronic medical record necessary to conduct this research.

***Financial support.*** This work was supported by the National Institute of Allergy and Infectious Diseases (grants T32 AI060530 and K24 AI134413 to E. H. G.), the Bill and Melinda Gates Foundation (grant OPP1105071), and the University of California San Francisco-Gladstone Institute of Virology and Immunology Center for AIDS Research (CFAR) Implementation Science Working Group, a National Institutes of Health–funded program (grant P30 AI027763).

***Potential conflicts of interest.*** The authors: No reported conflicts of interest. All authors have submitted the ICMJE Form for Disclosure of Potential Conflicts of Interest.
