# Supplementary material for: Longitudinal Care Cascade Outcomes Among People Eligible for Antiretroviral Therapy Who Are Newly Linking to Care in Zambia: A Multistate Analysis
Source: Clin Infect Dis. 2020 Mar 16;71(10):e561–70. doi: 10.1093/cid/ciaa268 (PMC7744998; doi:10.1093/cid/ciaa268)
Supplement: ciaa268_suppl_Supplementary_Appendix [file ciaa268_suppl_supplementary_appendix.docx]

**S1 APPENDIX**

**Multistage Sampling Approach and Derivation of Sampling Weights**

We undertook a multistage sampling approach in order to obtain population-representative estimates of patient outcomes across 64 facilities affiliated with the Centre for Infectious Diseases Research in Zambia (CIDRZ). In the first stage, we generated 12 facility strata that were defined by province and facility type (i.e., urban, rural, hospital). We then took a stratified sample of these facilities with the probability of selection being proportional to the size of the facility, selecting a minimum of 2 and up to a maximum of 10 facilities in each strata. This resulted in selecting 32 of the 64 total facilities that would yield results representative for all clinics despite differences in the size and number of clinics within each strata.

In the second stage of sampling, we enumerated patients who linked to care between April 1, 2014 and July 31, 2015 but who were lost to follow-up (LTFU) (defined as being at least 90 days late for the last scheduled visit or more than 180 days without any visit) as of July 31, 2015 at each of the selected facilities. Among the LTFU at each facility, we then selected a simple random sample of patients for tracing. Selected patients were then traced using a combination of chart review, phone calls, and in-person visits within the community in order to ascertain their current vital status (i.e., alive or dead), care status (i.e., out of care, in care at new clinic), and the associated dates using structured questionnaires.

In order to incorporate the updated outcomes that were ascertained through tracing, we derived sampling weights meant to ensure that the successfully traced patients were representative of the overall LTFU population despite any differences that arose due to differences in the number and size of clinics sampled per strata as a result of the multistage sampling schema. These sampling weights incorporated the probability of the facility being selected, the probability of being selected for tracing (if LTFU), and the probability of having outcomes successfully ascertained from tracing (if selected for tracing). For analyses stratified by patient subgroup, sampling weights were recalculated (i.e., recalibrated) after first restricting the dataset to that subgroup^1^.

**Table S1: Proportion of Patients in Each Care State at a given timepoint since Linkage to Care, n=23,227**

| Proportion of Patients in Each Care State at a given timepoint since Linkage to Care, n=23,227 | | | | | |
| --- | --- | --- | --- | --- | --- |
|  | 7 days | 30 days | 90 days | 180 days | 365 days |
| In Care on ART | 11.0 (10.0-12.1) | 47.4 (45.7-49.3) | 61.4 (59.3-63.6) | 63.1 (60.7-65.4) | 61.8 (58.5-64.9) |
| Reengaged on ART | 0.0 (0.0-0.0) | 0.0 (0.0-0.0) | 0.0 (0.0-0.0) | 1.2 (1.0-1.4) | 6.1 (5.5-6.7) |
| LTFU after ART | 0.9 (0.6-1.3) | 3.7 (2.7-4.8) | 7.2 (6.1-8.7) | 11.2 (9.2-13.2) | 8.5 (6.0-11.0) |
| Transfer after ART | 0.0 (0.0-0.0) | 0.3 (0.2-0.3) | 1.6 (1.0-2.5) | 2.8 (2.1-3.8) | 7.3 (5.0-10.3) |
| Died after ART | 0.1 (0.0-0.3) | 0.4 (0.2-0.6) | 1.7 (1.2-2.3) | 2.9 (2.1-3.9) | 3.9 (2.8-5.0) |
| In Care prior to ART | 78.4 (76.8-80.2) | 36.3 (34.6-37.9) | 14.2 (12.7-15.6) | 4.5 (3.7-5.5) | 1.3 (0.8-2.0) |
| LTFU prior to ART | 9.2 (7.5-10.9) | 10.9 (9.3-12.8) | 11.4 (9.7-13.3) | 10.6 (8.6-12.7) | 6.7 (4.6-8.8) |
| Transfer prior to ART | 0.0 (0.0-0.0) | 0.3 (0.1-0.7) | 0.8 (0.3-1.5) | 1.1 (0.6-1.9) | 1.4 (0.8-2.3) |
| Died prior to ART | 0.3 (0.1-0.7) | 0.8 (0.3-1.3) | 1.7 (1.1-2.5) | 2.6 (1.6-3.7) | 3.0 (1.9-4.2) |
|  |  |  |  |  |  |
| Composite Outcomes |  |  |  |  |  |
| ART Initiation | 12.0 (10.9-13.2) | 51.7 (49.8-53.5) | 71.9 (70.1-74.1) | 81.1 (78.8-83.4) | 87.6 (85.3-89.9) |
| In Care and on ART (All) | 11.0 (10.0-12.1) | 47.6 (45.9-49.6) | 63.0 (60.9-65.2) | 67.0 (64.6-69.3) | 75.2 (72.0-78.3) |
| LTFU | 10.1 (8.4-11.7) | 14.6 (12.8-16.6) | 18.6 (16.9-20.6) | 21.8 (19.3-24.4) | 15.2 (12.1-18.1) |
| Transfer | 0.0 (0.0-0.0) | 0.6 (0.3-1.0) | 2.5 (1.6-3.5) | 3.9 (3.0-5.1) | 8.7 (6.3-11.7) |
| Died | 0.4 (0.1-0.8) | 1.1 (0.7-1.7) | 3.4 (2.6-4.3) | 5.5 (4.3-6.9) | 6.9 (5.4-8.4) |

Footnote: All estimates are from multi-state models. Composite Outcomes Definitions: ART Initiation=ART in care [state 5], LTFU after ART [state 6], reengaged on ART [state 7], transferred on ART [state 8], and died after ART [state 9]; In Care and on ART=ART in care [state 5], reengaged on ART [state 7], and transferred on ART [state 8]; LTFU=LTFU prior to ART [state 2] and LTFU after ART [state 6]; Transfer=transfer prior to ART [state 3] and transfer after ART [state 8]; and Died=died prior to ART [state 4] and died after ART [state 9]. Abbreviations: ART=antiretroviral therapy; LTFU=lost to follow-up

**Table S2 Proportion of Patients in Each Care State after ART Initiation or Loss to Follow-Up**

| Proportion of Patients in Each Care State after ART Initiation or Loss to Follow-Up | | | |
| --- | --- | --- | --- |
|  | 90 days | 180 days | 365 days |
| ART Initiation, n=17,718 | 0.0 (0.0-0.0) | 0.0 (0.0-0.0) | 0.0 (0.0-0.0) |
| In Care on ART | 81.1 (78.6-83.4) | 74.6 (72.0-77.1) | 68.3 (64.7-71.7) |
| Reengaged on ART | 0.0 (0.0-0.0) | 2.3 (2.0-2.6) | 8.5 (7.7-9.6) |
| LTFU after ART | 13.3 (11.1-15.6) | 14.7 (12.3-17.4) | 8.8 (5.9-11.8) |
| Transfer after ART | 3.0 (2.0-4.4) | 4.5 (3.3-6.0) | 9.4 (6.6-12.9) |
| Died after ART | 2.7 (1.9-3.6) | 4.0 (2.9-5.2) | 5.0 (3.7-6.4) |
|  |  |  |  |
| LTFU Prior to ART, n=3,275 |  |  |  |
| In Care on ART | 0.0 (0.0-0.0) | 9.8 (7.7-12.6) | 27.4 (21.8-34.0) |
| Reengaged on ART | 0.0 (0.0-0.0) | 0.0 (0.0-0.0) | 1.0 (0.6-1.6) |
| LTFU after ART | 0.0 (0.0-0.0) | 0.9 (0.5-1.4) | 2.3 (0.7-4.5) |
| Transfer after ART | 0.0 (0.0-0.0) | 0.1 (0.0-0.3) | 3.2 (0.2-9.3) |
| Died after ART | 0.0 (0.0-0.0) | 0.0 (0.0-0.1) | 0.4 (0.0-0.9) |
| In Care prior to ART | 0.0 (0.0-0.0) | 2.4 (1.7-3.2) | 6.7 (3.5-11.8) |
| LTFU prior to ART | 100 (100-100) | 82.7 (76.7-87.0) | 49.3 (37.2-59.7) |
| Transfer prior to ART | 0.0 (0.0-0.0) | 0.5 (0.0-1.3) | 2.7 (0.7-5.9) |
| Died prior to ART | 0.0 (0.0-0.0) | 3.5 (0.2-8.5) | 7.0 (1.7-13.3) |
|  |  |  |  |
| LTFU after ART, n=3,894 |  |  |  |
| Reengaged on ART | 0.3 (0.0-1.1) | 28.9 (24.0-34.5) | 54.0 (46.5-64.9) |
| LTFU after ART | 99.7 (98.9-100) | 67.4 (60.9-72.9) | 30.3 (17.5-39.3) |
| Transfer after ART | 0.0 (0.0-0.0) | 2.0 (0.3-5.3) | 12.9 (4.4-23.1) |
| Died after ART | 0.0 (0.0-0.0) | 1.7 (0.3-4.7) | 2.8 (0.8-6.3) |

Footnote: All estimates are from multi-state models. Abbreviations: ART=antiretroviral therapy; LTFU=lost to follow-up

**Table S3: Proportion of Patients in Composite Care States at One Year Stratified by Patient Subgroups**

| Proportion of Patients in Composite Care States at One Year Stratified by Patient Subgroups | | | |  |
| --- | --- | --- | --- | --- |
|  | In Care on ART | LTFU | Transfer | Died |
| Overall | 75.2 (72.0-78.1) | 15.2 (12.4-18.1) | 8.7 (6.3-11.9) | 6.9 (5.4-8.4) |
|  |  |  |  |  |
| Sex and Age |  |  |  |  |
| Female <25y | 83.6 (77.2-89.8) | 9.4 (4.2-16.6) | 7.3 (4.5-10.9) | 2.2 (0.7-4.1) |
| Female 25-35y | 76.2 (70.3-81.8) | 17.2 (11.4-23.0) | 7.3 (4.5-11.2) | 3.8 (2.0-6.1) |
| Female 35-50y | 79.8 (74.5-85.3) | 12.9 (8.1-18.1) | 4.1 (2.9-5.9) | 5.6 (3.2-8.7) |
| Female >50y | 86.2 (80.7-90.9) | 1.5 (0.5-2.7) | 7.9 (4.7-11.9) | 6.8 (3.6-10.6) |
| Male <25y | 74.6 (61.4-87.0) | 13.9 (3.5-26.0) | 4.0 (1.9-7.0) | 7.1 (1.6-15.1) |
| Male 25-35y | 81.0 (72.9-86.6) | 7.3 (4.1-13.2) | 14.8 (5.5-23.2) | 9.5 (5.0-15.6) |
| Male 35-50y | 78.9 (73.7-84.1) | 11.9 (7.5-16.5) | 12.2 (6.0-19.1) | 7.0 (4.7-9.8) |
| Male >50y | 75.2 (72.2-78.2) | 15.2 (12.2-18.3) | 8.7 (6.2-11.6) | 6.9 (5.4-8.5) |
| Enrollment CD4 Count |  |  |  |  |
| CD4 <200 | 75.7 (71.1-79.9) | 12.3 (8.6-16.7) | 12.3 (7.1-18.1) | 9.7 (7.3-12.2) |
| CD4 200-350 | 82.4 (76.9-87.9) | 11.4 (6.1-16.4) | 6.4 (4.7-8.3) | 3.8 (1.6-6.9) |
| CD4 350-500 | 77.3 (69.9-84.7) | 18.6 (10.9-25.8) | 3.8 (2.8-4.9) | 1.8 (0.8-3.3) |
| CD4 >500 | 73.9 (64.9-82.7) | 18.6 (9.6-27.5) | 7.8 (2.9-16.6) | 2.1 (0.4-4.8) |
| Enrollment WHO Stage |  |  |  |  |
| WHO Stage 1 | 80.6 (75.7-84.8) | 13.2 (8.9-17.9) | 9.7 (5.8-13.8) | 3.2 (2.0-4.6) |
| WHO Stage 2 | 78.2 (71.1-84.9) | 12.5 (6.5-20.0) | 6.2 (4.3-8.3) | 7.0 (3.5-11.1) |
| WHO Stage 3 | 68.1 (62.3-74.8) | 14.4 (8.8-20.0) | 6.1 (4.4-8.4) | 14.0 (9.0-19.7) |
| WHO Stage 4 | 70.4 (59.7-80.0) | 4.8 (0.9-9.8) | 8.2 (3.5-13.8) | 24.0 (14.5-33.9) |
| Marital Status |  |  |  |  |
| Single | 75.7 (71.2-80.0) | 12.3 (8.3-16.6) | 12.3 (6.9-17.8) | 9.7 (7.3-12.1) |
| Married | 82.4 (77.3-87.7) | 11.4 (6.6-16.3) | 6.4 (4.8-8.4) | 3.8 (1.4-6.7) |
| Divorced | 77.3 (69.9-84.9) | 18.6 (10.9-26.7) | 3.8 (2.8-4.9) | 1.8 (0.8-3.1) |
| Widowed | 73.9 (65.0-83.0) | 18.6 (8.8-28.1) | 7.8 (2.8-16.2) | 2.1 (0.5-5.0) |
| Education |  |  |  |  |
| No Education | 81.5 (75.9-87.1) | 7.7 (4.0-12.7) | 5.1 (3.6-6.8) | 7.5 (3.2-12.5) |
| Lower-Mid Basic | 75.4 (71.0-79.9) | 14.8 (10.4-18.9) | 6.3 (4.5-8.6) | 6.6 (4.5-9.0) |
| Secondary | 76.1 (70.9-81.4) | 13.8 (9.0-18.7) | 9.3 (5.5-13.9) | 7.9 (5.0-11.1) |
| College/University | 79.0 (70.6-87.5) | 13.0 (4.6-21.2) | 11.1 (3.5-20.8) | 5.8 (3.2-9.1) |
| Facility Type |  |  |  |  |
| Urban | 72.3 (67.9-76.9) | 18.3 (13.9-22.6) | 9.1 (5.3-13.5) | 6.3 (4.2-8.5) |
| Rural | 67.8 (62.4-73.1) | 21.9 (16.6-27.5) | 6.1 (3.7-9.0) | 8.1 (5.3-11.1) |
| Hospital | 82.4 (78.8-86.1) | 7.5 (4.7-10.4) | 9.1 (6.3-12.6) | 7.9 (5.6-10.5) |
| Province |  |  |  |  |
| Lusaka | 70.5 (66.0-75.0) | 20.2 (15.6-24.9) | 9.2 (5.2-14.0) | 6.1 (4.0-8.6) |
| Eastern | 83.2 (79.8-86.5) | 6.7 (4.2-9.3) | 6.6 (4.9-8.7) | 6.6 (4.7-8.8) |
| Southern | 79.2 (74.7-83.8) | 11.6 (7.3-16.0) | 8.7 (5.7-12.1) | 6.7 (4.8-8.9) |
| Western | 79.9 (73.7-86.3) | 8.5 (4.5-13.4) | 9.8 (5.4-16.5) | 11.1 (6.3-16.7) |

Footnote: All estimates are from stratified multi-state models. Composite Outcomes Definitions: In Care and on ART=ART in care [state 5], reengaged on ART [state 7], and transferred on ART [state 8]; LTFU=LTFU prior to ART [state 2] and LTFU after ART [state 6]; Transfer=transfer prior to ART [state 3] and transfer after ART [state 8]; and Died=died prior to ART [state 4] and died after ART [state 9]. Abbreviations: ART=antiretroviral therapy; LTFU=lost to follow.
